# Mild histological distortions in rat organs after a 14-day oral exposure to the slime extract of African giant land snails

**DOI:** 10.1016/j.toxrep.2024.101743

**Published:** 2024-09-19

**Authors:** Damilare E. Rotimi, Morayo E. Barnabas, Tobiloba C. Elebiyo, Amarachi B. Iroaganachi, Funmilayo A. Okeniyi, Oluwakemi J. Awakan, Musbau A. Akanji, Oluyomi S. Adeyemi

**Affiliations:** a Department of Biochemistry, Medicinal Biochemistry, Nanomedicine & Toxicology Laboratory, Omu-Aran, Nigeria; b Landmark University SDG 3 (Good Health and Well-being Research Group), Omu-Aran, Nigeria; c Department of Pharmacology and Pharmaceutical Sciences, Alfred E. Mann School of Pharmacy and Pharmaceutical Sciences, University of Southern California, Los Angeles, California, USA; d Department of Biochemistry, Confluence University of Science and Technology, Okene-Lokoja Rd, Osara, Kogi 264103, Nigeria; e Department of Animal Science, Landmark University, Omu-Aran, Nigeria; f Department of Biochemistry, Kwara State University, Malete, Kwara State, Nigeria; g Department of Biochemistry, Medicinal Biochemistry, Nanomedicine & Toxicology Laboratory, Bowen University, PMB 232, Iwo 232101, Nigeria; h Laboratory of Sustainable Animal Environment, Graduate School of Agricultural Science, Tohoku University, 232-3 Yomogida, Naruko-onsen, Osaki, Miyagi 989-6711, Japan

**Keywords:** Acute toxicity, *Archachatina marginata*, Medicinal biochemistry, Histopathology, Zootherapy

## Abstract

**Objective:**

Snail slime possesses various pharmacological activities that are becoming attractive for zootherapy, thereby necessitating the profiling of its safety and toxicity. Therefore, using OECD 425 guidelines, this study assessed the acute toxicity of *Archachatina marginata* slime extract and performed a histological analysis of the vital organs.

**Methods:**

Eighteen (18) Wistar rats were assigned randomly into three groups: control, 2000 mg/kg, and 5000 mg/kg bw slime extract. The dosing of the animals with 2000 mg/kg bw and 5000 mg/kg bw was done according to the limit test procedure, after which the animals were observed for 14 days. During the observation period, clinical and behavioral changes were recorded. The rats were euthanized after 14 days of monitoring, and their essential organs were excised for gross histological examination.

**Results:**

There was no mortality during the observation period, and the LD_50_ of *A. marginata* slime extract was determined to be greater than 5000 mg/kg bw. Although there were no behavioral alterations in the rats after oral exposure to the slime extract, the histological examination revealed mild cellular distortions in the rat organs. Furthermore, a preliminary chemical analysis of the slime extract revealed the presence of flavonoids and phenolics.

**Conclusion:**

*A. marginata* slime extracts may be grouped as low toxic substance based on the results obtained (LD_50_ > 2000 – 5000 mg/kg). However, the histological distortions in rat organs following acute oral exposure to the snail slime extract not only warrant further, in-depth toxicological investigations but also caution in its use for traditional medicinal purposes.

## Highlights

• Snail slime did not remarkably affect the haematology and kidney function.
• Snail slime did not also affect the lipid profile, oxidative stress, or inflammatory markers.
• Hepatotoxicity was noted but the toxicity was reversed.
• Mild inflammation and cellular degeneration in organs.
• The chemical characterization detected the presence of some antimicrobial peptides.

## Graphical Abstract

**Figure:**
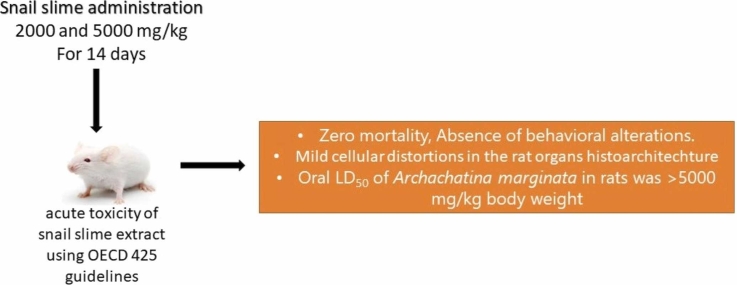

## 1 Introduction

A traditional medicine practice that employs natural products derived from animals to treat diseases is known as "zootherapy" [1]. These animal-based treatments can be formulated using the animal body parts themselves or any of their by-products. Compared to medicinal plants, research on therapeutic animals has yet to be fully developed [2]. Zootherapy, on the other hand, is gaining popularity as a viable alternative complementary therapy. For example, animal products from black caiman, ants, lions, hyenas, manatees, and earthworms have been used to treat abdominal pains, epilepsy, stroke, toothache, joint aches, rheumatism, backache, fever, malaria, hypertension, asthma, and allergic reactions [3], [4], [5], [6].

*Archachatina marginata,* a member of the phylum Mollusca and class Gastropoda, is edible meat with traditional medicinal applications. *A. marginata*, commonly known as the African giant land snail, has a high nutritional value; it is rich in protein and low in cholesterol and fat [7]. Moreover, the slime secreted by *A. marginata* and other snail species is a subject of ongoing exploration in traditional and folklore medicine. The slime contains mucin, a glycosaminoglycan (GAG) with anti-bacterial, anti-inflammatory, antioxidant, and anesthetic properties [8], [9], [10]. Mucin from the slime of *Achatina* species has been shown to possess gastroprotective properties, suggesting that it could be explored in the formulation of NSAIDs to minimize or remove their innate tendency to cause stomach mucosal damage [11], [12]. Collectively, snail slime holds prospects for drug and cosmetics development, but knowledge of its safety and toxicity profiling is limited. Therefore, this study assessed the acute oral toxicity of snail slime extract based on OECD 425 guidelines.

## 2 Materials and methods

Adult *A. marginata* (40) were procured from a local food market in Ibadan, Oyo State, Nigeria. Professor Lameed S. A. Gbolagade identified the snails in the Department of Wildlife and Ecotourism Management, University of Ibadan, Oyo State, Nigeria. The snails were housed in clean, well-aerated plastic cages. Eight snails were housed per cage to prevent overcrowding. Moist banana leaves or sand served as bedding. They were fed pawpaw, bananas, watermelon, pawpaw, and plantain, and water was intermittently sprinkled on the snails to keep them hydrated [13].

### 2.1 Extraction of slime

The extraction of *A. marginata* slime was done via the method described by Wiya et al. [14]. The snails were rinsed in distilled water to remove dirt and contamination before being placed in pre-warmed water at 32 °C to induce slime secretion. Immediately the snails came out of their shells; they were gently massaged for 1–5 min to enhance slime secretion. An equivalent amount of water was added to the slime, and the mixture was stirred for 1 h and centrifuged for 10 min at 3500 rpm. The supernatant was dispensed into clean tubes, lyophilized, and used for further experiments.

### 2.2 Preliminary chemical analysis the slime extract

The protein content of the slime extract was quantified using the method described by Gornall et al. [15]. The flavonoid and phenolic contents of the slime extract were determined using the method described by Lok et al. [16].

### 2.3 Experimental animals

Eighteen (18) non-pregnant and nulliparous female Wistar rats, aged six weeks and weighing between 90 and 110 g, were obtained from the Department of Biochemistry, University of Ilorin, Ilorin, Kwara State. They were transported to the animal holding facility of the Department of Biochemistry at Landmark University, Omu-Aran, Kwara State. The rats were acclimatized for seven days before the commencement of treatment. The animals were housed in clean and well-aerated cages and provided constant food and water throughout the study. The handling of the animals was consistent with Landmark University’s policy on animal use and ethics.

### 2.4 Evaluation of acute oral toxicity of the slime extract

Acute oral toxicity testing of snail slime aqueous extract was evaluated using the limit test of the OECD 425 guidelines [17]. The limit test was carried out by first dosing six Wistar rats with 2000 mg/kg body weight of the snail slime extract. The test animals were observed initially for the first 30 minutes, then for the next 4 hours at 30-minute intervals, and finally for the next 14 days. Observations such as changes in the skin, feces, urine, sleep, respiration, tremors, and itching were recorded. Following the absence of mortality after 14 days of exposure to 2000 mg/kg body weight of slime extract, a second limit test of 5000 mg/kg body weight was performed on a different group of rats. On the 15th day following acute exposure to the slime extract, the animals were euthanized under anesthesia using halothane. The organs were excised, weighed, and stored in 10 % formalin for histological examinations. However, they fasted for 12 h before euthanasia.

### 2.5 Histological evaluation

Representative samples of the rat kidneys, liver, ovaries, brain, stomach, heart, and spleen were fixed in buffered neutral formalin (BNF) and processed for histological examination as described by Owumi et al. [18]. Trimmed tissues were dehydrated in alcohol and dipped in xylene to remove the alcohol. The tissues were fixed in molten paraffin. The tissue blocks were then sectioned at 3–5 microns, floated in 20 % alcohol, and stained with hematoxylin and eosin (H & E) stain. The stained slides were left to dry and then viewed for pathological changes under a light microscope with x10 and x40 magnification. Photomicrographs were taken using a camera-mounted light microscope (AM-Scope 500), giving a micrographic representation of the various pathological observations.

### 2.6 Statistical analysis and data presentation

Statistical analysis was carried out using Graphpad Prism 9.0 software package (San Diego, California USA). The experimental values are presented as average of replicates ± standard deviation (SD).

## 3 Results

### 3.1 Preliminary chemical analysis of the slime extract

The *A. marginata* slime extract was rich in protein content; as shown in Table 1, 100 mg of the slime had 36.22 ± 1.15 mg of protein. In addition, preliminary chemical analysis for secondary metabolites revealed that the extract had flavonoids and phenolics. However, the phenolic content of the extract was low per 100 mg sample. As shown in Table 1, the flavonoid content of the slime extract was 6.41 ± 0.10 mg quercetin equivalent (QE) per 100 mg of the sample. For the phenolics, it was 0.837 ± 0.03 mg gallic acid equivalent (GAE) per 100 mg of the slime extract.

**Table 1:** Preliminary chemical analysis of the contents of *A. marginata* slime extract.

**Bioactive component**	**Composition (mg/100 mg)**
**Protein content**	36.22 ± 1.15
**Flavonoids**	6.41 ± 0.10
**Phenolics**	0.837 ± 0.03

Each value is a mean of three replicates ± SD.

### 3.2 Clinical and behavioral changes after acute exposure to *A. marginata* slime extract

After exposure to the slime extract, the animals were keenly observed for the first 30 min and then for the next 4 h at 30-min intervals in accordance with OECD 425 guidelines. For the next 14 days, the animals were monitored daily. Throughout the study, no clinical or behavioral changes were observed in animals exposed to either 2000 mg/kg bw or 5000 mg/kg bw slime extract when compared to the control group (Table 2). In addition, no mortality was observed throughout this study.

**Table 2:** Parameters observed at 30 min, 4 h, 24 h and once daily during the 14-day acute toxicity study of *A. marginata* slime extract in Wistar rats.

Parameters	Control	Slime (2000 mg/kg bw)	Slime (5000 mg/kg bw)
Fur and skin	Normal	Normal	Normal
Eyes	Normal	Normal	Normal
Saliva	Normal	Normal	Normal
Respiration	Normal	Normal	Normal
Urination (color)	Normal	Normal	Normal
Feaces consistency	Normal	Normal	Normal
Mobility	Normal	Normal	Normal
Sleep	Normal	Normal	Normal
Convulsions and tremors	Not found	Not found	Not found
Itching	Not found	Not found	Not found
Diarrhea	Not found	Not found	Not found
Survival rate	100 %	100 %	100 %

### 3.3 Histopathology of rat organs after exposure to *A. marginata* slime extract

**Fig. 1:**
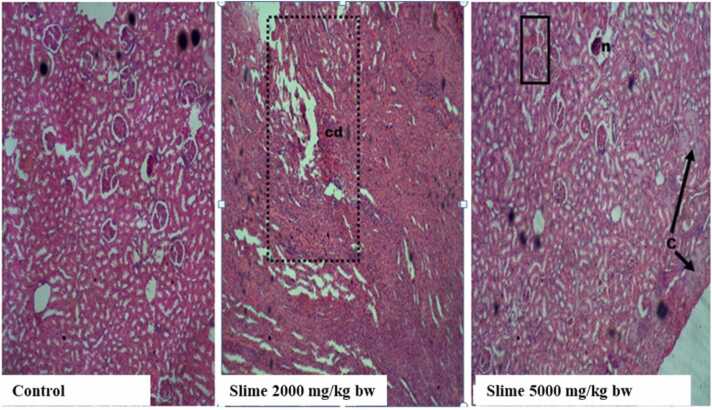
Representative photomicrographs of rat kidney following acute oral exposure to the slime extract of *Archachatina marginata*. (H & E, x10). The control shows no observable lesion or cellular changes, the glomeruli appeared normal with adequate capsular spaces. Slime 2000 and 5000 mg/kg bw presents with severe to mild degenerative changes (cd) and necrotic areas (n) which can be deduced from the overly large capsular spaces. The glomeruli were hemorrhagic and appeared edematous. Some mild fibrosis and peri-tubular inflammatory cells were also observed.

**Fig. 2:**
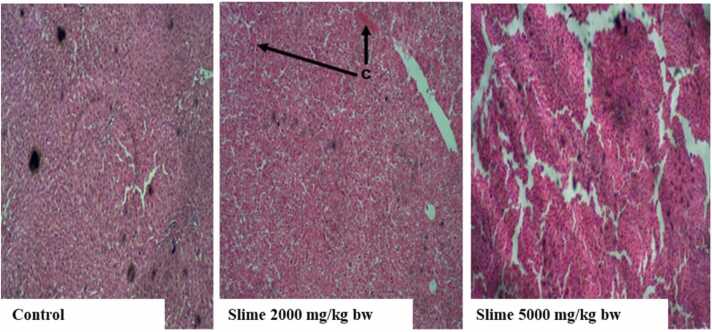
Representative photomicrographs of rat liver following acute oral exposure to the slime extract of *Archachatina marginata*. (H & E, x10). The control showed no observable hepatic damage. Slime 2000 mg/kg bw showed evidence of mild necrosis of the hepatocytes (c) with no significant cellular damages. Slime 5000 mg/kg bw showed evidence of moderate architectural cellular distortions, cellular damages and hepatocellular degeneration.

**Fig. 3:**
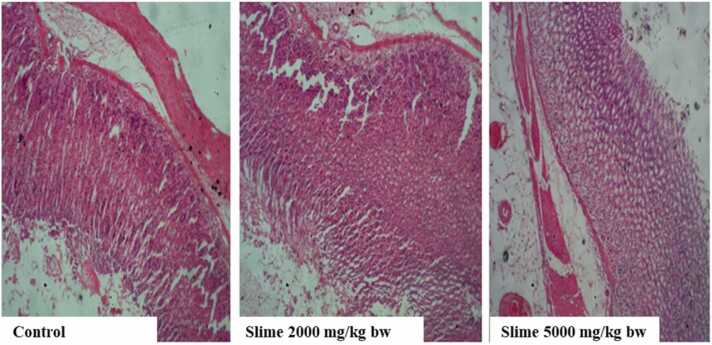
Representative photomicrographs of rat stomach following acute oral exposure to the slime extract of *Archachatina marginata*. (H & E, x10). Comparative observation across the micrographs shows well outlined arrays of normal gastrointestinal tissues with no significant observable cytoarchitectural distortion or any toxicological effects in the experimental groups.

**Fig. 4:**
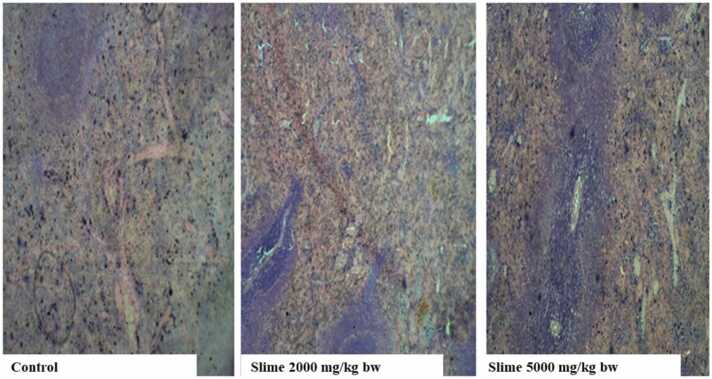
Representative photomicrographs of rat spleen following acute oral exposure to the slime extract of *Archachatina marginata*. (H & E, x10). The control showed well-outlined array of normal tissue without any observable cytoarchitectural distortion. Slime 2000 and 5000 mg/kg bw showed mild degenerative changes.

There were no visible lesions or cellular changes in the rat kidney in the control group (Fig. 1), and the glomeruli appeared normal with adequate capsular spaces. However, mild to severe degenerative changes and necrosis were observed in the rat kidneys after acute oral exposure to 2000 and 5000 mg/kg bw of the slime extract. The glomeruli of the rat’s kidney were hemorrhagic and appeared edematous. Mild fibrosis and peri-tubular inflammatory cells were also observed in the kidneys of the rats. The liver histology of the control animals showed no visible hepatic damage, while the livers of animals exposed to 2000 mg/kg bw of the slime extract showed evidence of mild necrosis of the hepatocytes. However, there was no significant cellular damage to the hepatocytes (Fig. 2). The livers of the animals exposed to 5000 mg/kg bw of the slime extract showed evidence of moderate architectural cellular distortions, cellular damages, and hepatocellular degeneration. There were no significant cytoarchitectural distortions in the gastrointestinal tissues of the animals in all the experimental groups (Fig. 3). The histological evaluation of the spleen from the control group also showed a well-outlined array of normal tissue without any observable cytoarchitectural distortion (Fig. 4). However, the spleens of the animals given 2000 and 5000 mg/kg bw of slime extract showed mild cellular degeneration.

**Fig. 5:**
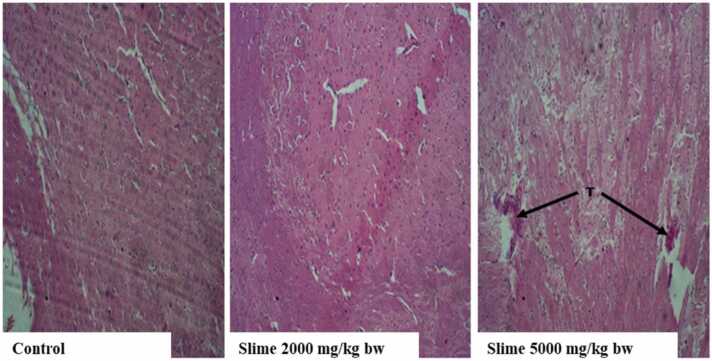
Representative photomicrographs of rat brain following acute oral exposure to the slime extract of *Archachatina marginata*. (H & E, x10). The control and Slime 2000 mg/kg bw showed no visible lesion. Slime 5000 mg/kg bw showed mild chronic inflammatory cells which consisted of multinucleated cells (T). There were a few areas of necrosis with a cellular debris.

**Fig. 6:**
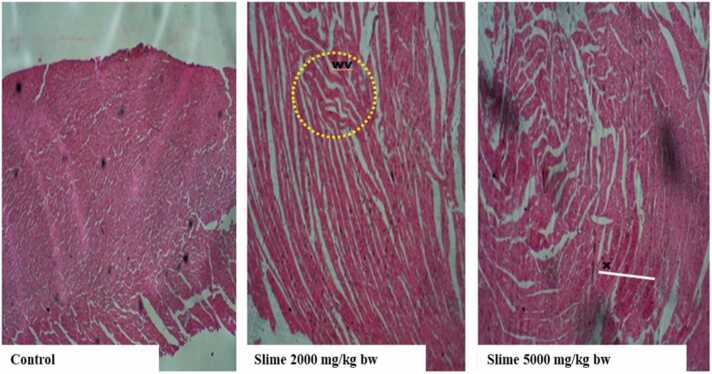
Representative photomicrographs of rat heart following acute oral exposure to the slime extract of *Archachatina marginata*. (H & E, x10). The control had no observable cellular damages, degenerative changes, or necrosis. Slime 2000 and 5000 mg/kg bw showed mild to severe Wavy Cardiac Muscles (wv) cell with mild necrotic (x) cells.

The brain tissues of the control animals and the animals exposed to 2000 mg/kg bw of the slime extract showed no visible lesions (Fig. 5). Still, the brain tissues of animals administered 5000 mg/kg bw were characterized by chronic infiltration of inflammatory cells, especially multinucleated cells (T). A few areas of necrosis with cellular debris were also observed in the brain tissues of the animals exposed to 5000 mg/kg bw of the extract. The heart histology of the control group had no observable cellular damages, degenerative changes, or necrosis, while the animals exposed to 2000 and 5000 mg/kg bw of the extract showed mild to severe Wavy Cardiac Muscle (wv) cells with mild necrotic (x) cells (Fig. 6).

**Fig. 7:**
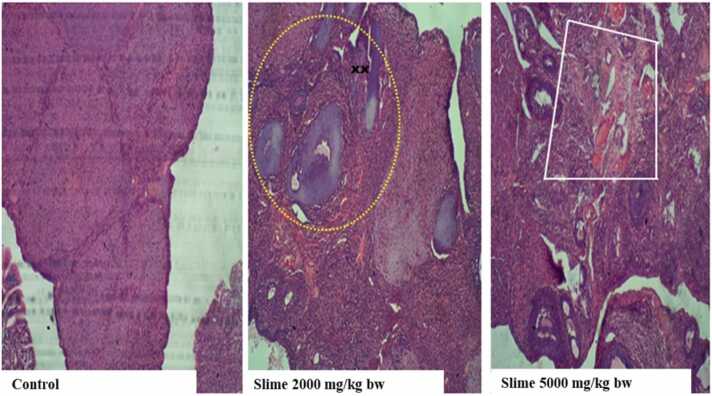
Representative photomicrographs of rat ovary following acute oral exposure to the slime extract of *Archachatina marginata*. (H & E, x10). The control showed dilated blood vessel, inter-stroma adipose connective tissue, normal oviduct and primordial follicle in various stages of developments. Slime 2000 and 5000 mg/kg bw showed atrophied ovarian cells (xx), some wasted follicle and mild degenerative changes as well as some mild fibrosis.

The histological examinations of the ovaries of the control animals showed normal blood vessel dilation, inter-stromal fatty connective tissue, a normal oviduct, and a primordial follicle in various stages of development. The animals exposed to varying concentrations of the slime extract showed atrophied ovarian cells (xx), wasted follicles, mild degenerative changes, and mild fibrosis (Fig. 7).

## 4 Discussion

The slime extract of *A. marginata* had an appreciable protein content, consistent with a previous report [19]. More so, the protein content of *A. marginata* slime has been implicated as partly responsible for its antimicrobial and wound-healing properties [20]. In addition, the presence of flavonoids and phenolics in the slime extract of *A. marginata* supports its acclaimed medicinal value [8], [9], [10]. Flavonoids and phenolics are secondary metabolites mostly found in plants, but emerging evidence shows that animals could derive these metabolites from plant material in their diet or through other biochemical metabolism [21]. Interestingly, *A. marginata* feeds on fruits, green vegetables, and other leaves (pawpaw and banana leaves) known to have large amounts of polyphenols, especially flavonoids [22]. Therefore, it is plausible that the flavonoids and phenolics came from the plant-based diets. The pharmacological activities of snail slime may be attributed to the presence of these secondary metabolites. Flavonoids and phenolics possess anti-inflammatory, anti-oxidant, hepatoprotective, and anti-cancer activities [23].

Furthermore, no mortality was recorded throughout this experimental study, and the oral LD_50_ of the slime of *A. marginata* in rats was estimated to be above 5000 mg/kg bw. Using the globally harmonized classification systems, chemical substances, including natural products, can be grouped into five classes based on their LD_50_. Since the slime extract of *A. marginata* has an oral LD_50_ > 5000 mg/kg bw in rats, it can therefore be classified as a category five substance with relatively low acute toxicity [24]. Morphological and behavioral changes after exposure to a substance are often indicative of toxicity [17], [25]. However, acute oral exposure to 2000 and 5000 mg/kg bw of the slime extract did not cause any notable clinical or behavioral changes in the rats. Their sleep and mobility patterns were normal, and there were no signs of respiratory distress, diarrhea, itching, convulsions, or tremors. There was no discoloration of the fur and skin, urine, or eyes. The histopathological examination of the rat organs showed no lesions, inflammation, or degenerative changes in the control group. However, the group administered 5000 mg/kg bw had mild degenerative changes, inflammation, and mild necrosis in the liver, kidney, spleen, brain, heart, and ovaries. The stomach histology of the animals exposed to the different concentrations of the slime extract showed no observable cytoarchitectural changes. Although some of our findings are consistent with previous research suggesting that *A. marginata* slime extract may have gastroprotective properties, organ lesions in the other organs caused by acute exposure to the extract are a cause for concern [11], [26].

## 5 Conclusion

Collectively, the data showed that *A. marginata* slime extracts may be grouped as low toxic substance (LD_50_ >2000 – 5000 mg/kg bw). However, the histological distortions in rat organs following acute oral exposure to the snail slime extract not only warrant further, in-depth toxicological investigations but also caution in its use for traditional medicinal purposes. In addition, a future investigation should explore the chemical characterization of the slime extract of *A. marginata* to identify bioactive constituents.

## Ethics approval and Consent to participate

The care, use and treatment of the rats were carried out as described and approved by the Landmark University Ethical Committee with approval number LUAC/2021/002A on 23rd November, 2021.

## Funding

This research did not receive any specific grant from funding agencies in the public, commercial, or not for- profit sectors.

## CRediT authorship contribution statement

**Tobiloba C. Elebiyo:** Project administration, Resources, Software, Supervision. **Morayo E. Barnabas:** Conceptualization, Formal analysis, Funding acquisition, Project administration, Resources, Supervision, Writing – original draft. **Funmilayo A. Okeniyi:** Conceptualization, Formal analysis, Funding acquisition, Investigation. **Damilare Rotimi:** Writing – review & editing, Writing – original draft, Validation, Funding acquisition, Formal analysis, Data curation. **Amarachi B. Iroaganachi:** Software, Supervision, Validation. **Musbau A. Akanji:** Supervision, Validation, Writing – review & editing. **Oluwakemi J. Awakan:** Conceptualization, Funding acquisition, Investigation, Methodology. **Oluyomi S. Adeyemi:** Conceptualization, Data curation, Resources, Software, Writing – original draft, Writing – review & editing.

## Declaration of Competing Interest

The authors declare that they have no known competing financial interests or personal relationships that could have appeared to influence the work reported in this paper.

## Data Availability

Data will be made available on request.
